# An Internet of Things Framework for Monitoring Environmental Conditions in Livestock Housing to Improve Animal Welfare and Assess Environmental Impact

**DOI:** 10.3390/ani15050644

**Published:** 2025-02-23

**Authors:** Giorgio Provolo, Carlo Brandolese, Matteo Grotto, Augusto Marinucci, Nicola Fossati, Omar Ferrari, Elena Beretta, Elisabetta Riva

**Affiliations:** 1 Department of Agricultural and Environmental Sciences, University of Milan, 20133 Milan, Italy; omar.ferrari@unimi.it (O.F.); elena.beretta5@studenti.unimi.it (E.B.); 2 Department of Electronics, Information and Bioengineering, Politecnico di Milano, 20133 Milan, Italy; carlo.brandolese@polimi.it; 3 IBT Systems, 20133 Milan, Italy; matteo.grotto@ibtsystems.it (M.G.); augusto.marinucci@ibtsystems.it (A.M.); nicola.fossati@ibtsystems.it (N.F.)

**Keywords:** animal welfare, gas concentrations, ammonia emissions, ventilation

## Abstract

Devices for assessing the quality of animal environments are important for maintaining production animals, thus improving animal well-being and mitigating pollutant emissions. Therefore, an IoT system was developed and preliminarily assessed across various livestock housing types, including those for pigs, dairy cows, and rabbits. This system measures and transmits key parameters, such as ambient temperature; relative humidity; light intensity; sound pressure; levels of carbon dioxide, ammonia, and hydrogen sulfide; and particulate matter and volatile organic compound concentrations. These data are sent from the sensors to a gateway and then displayed on a dashboard for monitoring. A preliminary evaluation of the system’s performance in controlled conditions revealed that the device’s accuracy and precision were within 2.7% and 3.3% of the measured values, respectively. The system was deployed in three case studies involving rabbit, pig, and dairy cow farms. The results demonstrated its effectiveness in assessing pollutant emissions and identifying critical situations where gas concentrations exceeded threshold levels, thus posing a risk to the animals. By systematically applying this technology on livestock farms to obtain a detailed understanding of the microclimatic and air quality conditions in which the animals live, animal welfare can be significantly improved.

## Simple Summary

Microclimate conditions and air quality in animal housing facilities are key aspects of animal welfare and require appropriate monitoring devices. For this reason, a multi-parameter device was developed that can be easily installed in housing thanks to its small size and rechargeable battery operation. Using these devices in different types of farms allowed us to verify their operational characteristics and functionality, particularly the following: the lack of uniform ventilation with high ammonia concentrations in a rabbit farm; the correlation between temperature, ammonia, and carbon dioxide concentrations in a piggery; and the trend in ammonia emissions in a dairy cattle farm. Using these devices to monitor environmental conditions in livestock farms is essential for ensuring adequate animal welfare conditions.

## 1. Introduction

Appropriate environmental conditions in livestock housing are essential for ensuring animal welfare and productivity [1]. Currently, the attention paid to environmental factors has been increasing, particularly regarding climate change, as increased temperature, particulate matter, and ammonia (NH_3_) concentrations can negatively affect the animals [1,2,3,4]. The main factors considered are temperature, humidity, and, in some cases, air velocity, but the environmental conditions in livestock housing should also include factors such as light intensity, gas concentrations, and noise levels [5]. In some cases, the limitations and optimal levels are defined for these factors and it is well known that they can affect animal welfare [6].

For example, the EFSA considers air quality to be inadequate for pigs when carbon dioxide (CO_2_) levels are above 3000 ppm, carbon monoxide levels are above 10 ppm, hydrogen sulfide levels are above 0.5 ppm, and/or NH_3_ levels are above 10 ppm, with consideration also required for dust and high airspeed. The same limits for NH_3_, CO_2_, and hydrogen sulfide apply to dairy cows [7,8].

Moreover, some gasses emitted by livestock farms have significant environmental impacts. For instance, NH_3_ is a gas that causes acidification and eutrophication and combines with other substances to form PM_2.5_. To reduce these harmful environmental impacts, member states of the European Union must limit their NH_3_ emissions in accordance with the National Emissions Ceilings Directive (2016/2284/EU).

Measuring gas concentrations in livestock farms is a challenging task for different reasons, including the following: the spatial and temporal variability of the processes that produce emissions; the measuring equipment presenting uncertain results; and the methodology possibly not considering all aspects, introducing simplification and additional uncertainties [9,10,11]. For these reasons, devices that monitor environmental conditions with different working principles have previously been used in livestock farms [12,13].

The concentration of gasses such as NH_3_, H_2_S, and CO_2_ can be measured with different technologies and approaches. Some techniques predict the cumulative measurement of concentrations and allow average values to be obtained over the exposure time frame. For continuous monitoring of gas concentrations in barns—required for checking adequate conditions for animals—techniques that allow rapid responses that can be used over long periods of time need to be adopted [14]. Among these, analyzers based on Fourier transform infrared (FTIR), photoacoustic infrared spectroscopy, or cavity ring-down (CRDS) principles can provide accurate measurements of gas concentrations in the barn. However, the cost of the equipment and in some cases of maintenance limits their use outside a research framework—these instruments are too costly and require too much maintenance for widespread, continuous on-farm use [14,15].

Alternatively, low-cost sensors using principles like optical absorption, electrochemicals, and metal oxide semiconductors are less accurate, but their affordability and ability to monitor multiple points simultaneously offer significant advantages [14,15,16,17]. Moreover, the integration of these sensors into an IoT device can enable real-time monitoring at different points in the housing facilities to continuously provide useful information for both researchers and farmers, and they can be a component of an automatic control system (e.g., by controlling ventilation) and early warning for environmental conditions that are unsuitable for animal and operator welfare.

Only a few studies can be found in the literature that have investigated the application of low-cost sensors to monitor relevant gas concentrations in livestock housing [15]. Furthermore, Danev et al. [17] highlighted the importance of developing multi-sensor devices to monitor the environmental conditions in barns. They propose to integrate sensors for ammonia, hydrogen sulfide, carbon dioxide, particulate matter, total volatile organic compounds (TVOC), temperature, humidity, and pressure to offer a holistic view of air quality conditions.

In precision livestock farming, a monitoring device for environmental conditions must be able to integrate recordings with other data collected in livestock housing and those from the animals using wearable sensors or other devices [18]. Leliveld et al. [19] demonstrated the feasibility of this approach and developed an IoT system for dairy cow farms. However, it became clear that a suitable multipurpose device was needed to monitor environmental conditions in livestock housing [20]. As a result, an IoT device that records the main climatic parameters and gas concentrations was developed and installed in different livestock farms to record the environmental conditions and assess their adequacy in meeting livestock requirements.

Few studies have verified the use of low-cost multi-sensor devices to assess the uniformity of environmental factors under controlled and operational conditions, including the trends in variables over time and quantifying emissions. However, the use of such devices can contribute significantly to improving the animal welfare and environmental sustainability of livestock farms. Therefore, the purpose of this study is to provide a contribution in this direction, with the specific aim to (1) describe the architecture of the developed device and its connection to an IoT environment; (2) assess the device’s performances in a controlled environment; (3) and to evaluate its practical use in three livestock housing conditions.

## 2. Materials and Methods

The device used in the study had some specific characteristics, such as its integration of the main environmental measure for assessing air quality in relation to animal welfare, the flexibility of the power supply (battery or wired), and its IoT capability. The device characteristics were described and its performances were assessed in controlled conditions. Furthermore, the operating performance and application of the device in three case studies were assessed and reported on to illustrate the applicability of the system to different livestock housing environments.

The first case was a rabbit farm, where the objective was to evaluate the trend and uniformity of environmental conditions in the housing in order to highlight critical zones in the area that might affect animal health. The second case study was a pig farm, where the device was used to analyze the daily and hourly fluctuations of gas concentrations, specifically in connection with management operations to identify correlations among variables and specific patterns during the day. The last case was a dairy cow farm, where the continuous collection of gas concentrations was used to estimate NH_3_ emissions from the barn to support the environmental assessment of the livestock.

### 2.1. Device for Environmental Monitoring

#### 2.1.1. Device Architecture for Environmental Monitoring

Environmental monitoring was carried out using a multi-sensor node developed by IBT Systems (Milano, Italy) in collaboration with the Department of Agricultural and Environmental Sciences of the University of Milan. The environmental sensor node, referred to as N11 in this study, is one of the several sensors initially developed for the integrated monitoring of dairy cattle farms [19] that was extended and adapted for other livestock. This device was derived from the evolution of the sensor technology used in a previous project [19] and was developed because a device with similar characteristics was not available, particularly regarding the integration of sensors for the measurement of environmental variables and the ability to operate with an internal battery. In addition, the device used is compatible with the IoT system implemented on some farms and therefore functional for ongoing research activities.

The N11 node is composed of four main parts:

- Main board: The main board hosts and connects all the components of the N11 node. In addition to these components, the main board also hosts a 128 Mbit SPI flash memory used to locally store sensor data. The goal was to provide a standalone (i.e., without gateway and internet connection) operating mode of the N11 board and/or offer a backup mechanism in the case of temporary network failure or connection unavailability. The mechanical design of the main board was co-developed with a custom plastic housing designed specifically for the node and target environment.
- Power supply unit: The power supply unit consists of rechargeable batteries and the charger control circuit, which is capable of operating both from a 12 V DC voltage source, typically generated via a commercial power adapter connected to AC mains, and via a solar panel with a 12 V backup battery. The internal batteries guarantee continuous operation for approximately 20/25 days.
- Processing unit: The processing unit is based on an ultra-low power system on a chip that provides significant processing resources (flash memory, volatile memory, and computational power) and integrates a dual-radio transceiver supporting both Bluetooth Low Energy (BLE) and custom sub-gigahertz modulation (RF868). Both radio interfaces are used by the N11 node, with BLE being limited to local communication with a mobile app for configuration and diagnostic purposes and the RF868 custom radio to data communication with the local gateway.
- Sensors: The sensors are either standard digital components (e.g., particle matter sensor) directly connected to the main board and the processing units through I2C or UART buses or custom expansion boards designed to condition the sensors’ analog output signals and connect them to the analog-to-digital converters integrated into the processing units.

**Figure 1:**
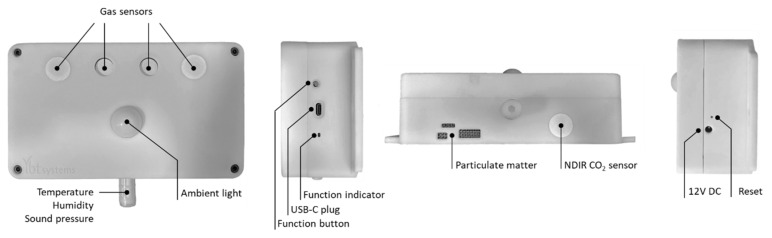
The device for environmental monitoring (N11) with enclosure.

Figure 1 shows the complete sensor node in its plastic case.

#### 2.1.2. Sensor Expansion Boards

The main board provides several interfaces dedicated to the connection of the sensors’ expansion boards, namely, four analog inputs, one UART bus, and one I2C bus. The designed sensors’ expansion boards are described as follows:

- Temperature, humidity, and sound sensor: This expansion board integrates the Sensirion (Stäfa, Switzerland) SHT30 temperature and humidity digital sensor and the Knowels (Itasca, IL, USA) SPW2430 analog MEMS microphone. The temperature and humidity sensor was connected to the processing unit through the I2C bus, while the microphone output was low-pass filtered and amplified with a gain of approximately 30 dB before being forwarded to the ADC of the processing units.
- Illumination sensor: This expansion board mainly provides I2C connectivity and mechanical support for the Vishay (Malvern, PA, USA) VEML7700 ambient light sensor, which provides a direct lux reading on the I2C bus.
- Electrochemical gas sensors: This expansion board is more complex than the ones described above and was primarily designed to host a wide variety of electrochemical sensors and to be easily pluggable into the main board to expose the active part of the sensors to the openings in the plastic case. The most sophisticated part of this expansion board consists of the analog signal conditioning frontend that is necessary to amplify and filter the electrochemical sensor current output signal. In fact, the output current is affected by a zero-level offset and is extremely low, in the order of 10–200 nA/ppm, depending on the specific gas. In addition to the sensor analog frontend, the expansion board also integrates a 1 Kbit non-volatile (EEPROM) memory with an I2C interface. This small memory is used to store the calibration parameters (offset and gain) of the specific gas sensors. Because of this memory and a standard 2.54 mm header connector, the electrochemical sensors can be calibrated using a specific host board and calibration firmware, which can store the calibration parameters on the EEPROM memory. As a result, the calibrated sensor and its host board can be removed from the calibration board and installed in the N11 sensor node.
- VOC sensor: A variant of the electrochemical sensor board was also designed to integrate the digital I2C Sensirion SGP41 VOC/NOx sensor.

In our experiments, the N11 node was equipped with the sensor listed in Table 1, which also outlines their main characteristics.

Some of the sensors need to be read as analog signals, while others provide a digital interface (Table 1). In the latter case, the measurement process is internally managed with the sensor logic, which only provides data at a certain frequency, selectable when the sensor is configured with the application firmware. For analog sensors, on the other hand, the measurement process is entirely under the control of the electronics and firmware of the N11 node.

Considering the nature of the physical phenomenon, the sensor characteristics, and the constraints on the transmission of data to the gateway, which is scheduled at regular 10 min intervals, the measurements are performed as follows:

- Temperature and humidity: Sensors are read every minute and averaged over the 10 min period.
- Electrochemical: Sensors are sampled every 10 s and averaged over each 10 min period.
- Sound pressure: The sensor is sampled every 10 s, and the maximum and average values are calculated for each 10 min period.
- CO_2_ and ambient light: Sensors are read every minute and averaged over the 10 min period.
- Particulate matter and VOC: Sensors are read every 15 min and sent to the gateway every 10 min, skipping intervals where the measure is not yet ready and available. Although it might not record very quick variations in the concentrations of these variables, a sampling period of 15 min was chosen as a compromise between frequent measurements and energy requirements when the device is battery operated, and the goal was to reach 3 weeks of continuous operation. However, the sampling frequency is a firmware parameter that can be easily set using a dedicated mobile application.

Every 10 min, the firmware collects all the available measures; computes the average, maximum, and minimum values; and composes a single binary-encoded compact packet to be stored on the N11 flash memory and transmitted to the gateway through the custom sub-gigahertz wireless channel.

#### 2.1.3. IoT Architecture

The N11 sensor nodes are part of a wider IoT architecture developed over the last four years within the context of the GALA project [19]. The architecture is structured as a typical IoT system and consists of heterogeneous sensor nodes (edge devices), a communication gateway (fog device), and a cloud infrastructure.

The communication between the sensor nodes and gateway is supported with a custom binary protocol over a physical 868 MHz channel with FSK/QPSK/BPSK modulations, depending on the desired transmission range. To minimize the energy consumption of the sensor nodes caused by packet collision, the application protocol is designed according to a time-division approach, where each node is assigned to a specific time slot in the 10 min period after an initial negotiation phase. Each slot has a one-second duration and is divided into an initial uplink phase, where the node transmits data to the gateway, followed by a short silence and downlink phase, where the node is configured in the receive mode and waits for possible data transmitted via the gateway. During the downlink phase, the node periodically receives a synchronization message that corrects the drift of the nodes’ clock with respect to the central time base of the gateway.

From the hardware point of view, the gateway is constituted using an industrial 4G modem and a custom receiver for the local radio 868 MHz channel. The custom radio receiver is based on the same processing unit of the nodes adapted to implement a serial interface used to forward the raw binary packets received by the radio module to the modem. The application protocol implemented over this serial interface is Modbus RTU, with the modem acting as the master device in charge of actively reading data from the radio receiver.

The gateway does not decode the content of the binary messages received from the sensor nodes through the radio receiver. Its role solely consists of packing individual messages into a larger binary message, which is then forwarded to the cloud infrastructure using the MQTT protocol. Furthermore, the gateway double buffers the messages, making it resilient to communication failures and power shortages. Incoming messages are first buffered in the RAM to deal with short-duration network failures. When the connection is re-established, buffered messages are sent to the cloud and removed from this buffer; if the connection problems persist, unsent messages are moved from the RAM buffer to a second larger buffer stored on the filesystem in flash memory. This second buffer also ensures that unsent messages are not lost in the case of power shortages. At each power-on process, the gateway checks for the presence of buffered messages on the filesystem; if present, it sends them to the cloud. It is worth noting that messages are timestamped by the nodes when they are generated, guaranteeing that delayed transmissions do not affect the association of messages to their time of generation.

The messages sent by the gateway are collected with an MQTT broker that unpacks the incoming messages into individual payloads, decodes such binary payloads, and generates a human-readable JSON message that is finally forwarded to the MQTT broker of the cloud application based on the Thingsboard v.3.2.2PE framework (ThingsBoard Inc., New York, NY, USA). This application collects and first stores the incoming messages into a structured database where data are organized into “assets” corresponding to different farms or installations, “devices” representing the sensor nodes, and “timeseries” associated with individual measurements. Once stored, each single message is forwarded into a set of “rulechains”, i.e., processing paths that can perform different kinds of operations depending on the device type and the specific time series. Such operations typically consist of computation over time on a single time series (e.g., average over the last 24 h) or a combination of the last measurements of multiple time series (e.g., the combination of temperature and relative humidity to compute THI). The results of such computations have the effect of creating new time series, which are then stored in the database along with the raw measurements from the field.

Finally, all the collected data are grouped according to different criteria (per farm, sensor node, measurement, etc.) and presented to the user via custom dashboards designed for specific purposes.

### 2.2. Calibration and Assessment of Device Performance

#### 2.2.1. Calibration Equipment

The CO_2_, H_2_S, and NH_3_ sensors need to be calibrated before their usage in the field [21]. For this study, the calibration equipment necessary for this process consisted of a complete N11 board and the mobile application. Furthermore, for the electrochemical sensors, an ad hoc plastic cap was designed and 3D printed. Such caps must be applied on the top of electrochemical sensors to convey a controlled air flow, exposing the sensors to a known gas concentration. On the other hand, calibrating the CO_2_ sensor does not require special additional equipment.

Once the hardware setup was completed, the mobile app was used to force the N11 node to perform one or two measurements at known gas concentration to compute sensor offset and gain and to instruct the N11 node to write such parameters in the EEPROM memory of the appropriate sensor expansion board.

#### 2.2.2. Calibration Procedure

Calibration is a necessary operation for certain sensors which can vary in their response over time despite having known calibration curves. In the case of the multi-parameter control unit used, a span calibration (two points) was performed for the NH_3_ and H_2_S electrochemical sensors. In the case of CO_2_, zero (offset) calibration only was carried out. The procedure was performed according to the datasheet of the sensors used.

For NH_3_ and H_2_S, the procedure involved the use of a cylinder with a known concentration of the gas and a dynamic dilution calibrator (ETG CAL G100, ETG Risorse e Tecnologia S.r.l, Turin, Italy) to obtain the desired concentrations. N_2_ was used as a carrier gas. The sensors, placed on the calibration board described above, were fitted with adapters connected to the dilution calibrator with 6 mm diameter PTFE tubes.

In addition, for NH_3_, a check of the actual concentration applied to the sensors was carried out with a gas analyzer (GT5000, Gasmet Technologies Oy, Vantaa, Finland) connected to the output of the sensor caps. CO_2_ calibration was carried out by placing the boards in ambient conditions. The reference concentration was obtained with GT5000 by measuring the same air.

The calibration system used necessarily introduced uncertainty due to the accuracy of cylinder concentration and gas analyzer (both 2% of the measured value). However, this uncertainty is lower than the accuracy of the calibrated sensors shown in Table 1.

**Figure 2:**
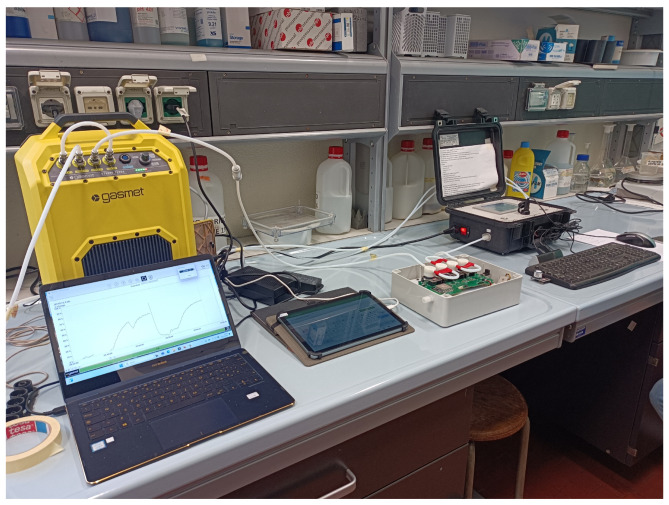
Calibration setup used for NH_3_ and H_2_S sensors.

The calibration setup is shown in Figure 2.

#### 2.2.3. Checking Control Unit Operation

The objective of these tests was to evaluate the use of the instrument under realistic operating conditions using a sensor set with known operation and nominal accuracy.

For this reason, the verification focused on the distribution of measurements made with different devices placed under the same operating conditions to assess repeatability.

No comparative tests were carried out for the device and other reference instruments in livestock buildings for two reasons: First, the barn environment is subject to environmental conditions (e.g., air flow) that can vary in both in space and over time; second, because of the different measurement principles of the gas analyzer and the device. The former is equipped with a small pump that collects air in a closed chamber to perform the measurements, while the electrochemical sensors of the device operate via absorption of the target gasses in natural air flow conditions. For the two measures to be comparable, the air to be characterized should be in a steady condition, both in terms of concentration and air flow.

Considering the accuracy of the sensors shown in Table 1, it is rather difficult to guarantee a steady-state condition with this level of accuracy, hence the in-lab approach that we have chosen.

The accuracy of the measurements made by the control units was subsequently verified by placing a set of 8 control units under the same measurement conditions and checking the variation in their responses.

For the H_2_S and NH_3_ electrochemical sensors, measurements were performed using the same equipment adopted for the calibration to obtain the measured values post-calibration. In the case of CO_2_, temperature, relative humidity, particulate matter, and sound pressure, the control units were placed in the same environment for 10 h and data were downloaded from the dashboard.

**Figure 3:**
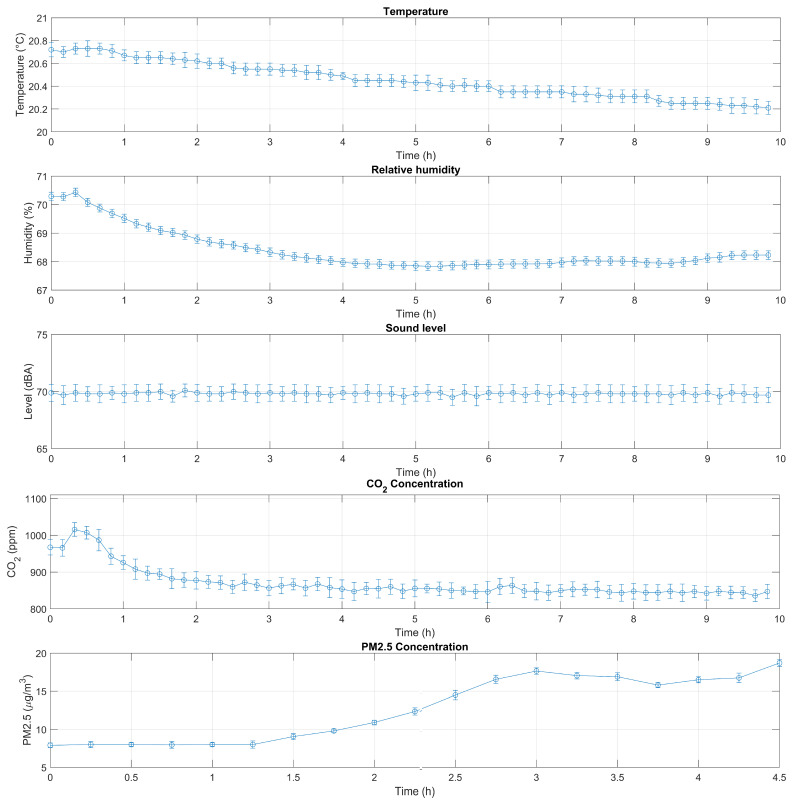
Average and standard deviations of the absolute error of the different sensor nodes at each measurement interval.

The average and standard deviations of the absolute error of the different sensor nodes at each measurement interval (i.e., every 10 min) were calculated to evaluate the different responses of the device and determine the comparability of the measurements, as shown in Figure 3.

Finally, these average and standard deviations were averaged over time for the entire experiment duration (Table 2).

As shown, both the average error and the average standard deviation are below 3.5%. These values are coherent with the characteristics of the sensors reported in Table 1.

The purpose of checking the devices under operating conditions was to verify that, once calibrated, they provided comparable results. Even in this case, the relative error between different sensors was lower than the accuracy of the sensors (Table 1).

Therefore, with the understanding that the accuracy of the measurements depends on the characteristics of the sensors, the measurements made by different devices under operating conditions can be considered comparable.

### 2.3. Study Cases

#### 2.3.1. Study of Environmental Condition Uniformity in a Rabbit Farm

**Figure 4:**
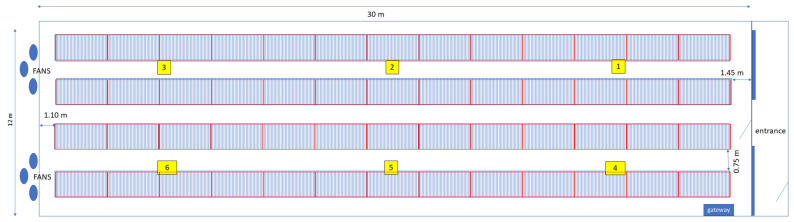
Scheme of the structure of the rabbit farm and position of the six N11 devices during the monitoring period, indicated by the yellow boxes. The numbers represent the ID of the devices.

The farm was situated in Volpago del Montello (TV). The monitored room was 12 m wide and 30 m long with a constant height of 4 m, hosting 4000 rabbits in 4 batteries of double cages. The farm had no natural illumination and did not use an artificial light program. The dry feed was delivered by an automatic system in each cage. The ventilation system was controlled by a central unit that adjusted the fan speed according to the preset temperature in the room. Six N11 devices were installed at the cage level. Figure 4 shows the building layout and device position. Monitoring was carried out from 14 March 2023 to 20 March 2023.

**Figure 5:**
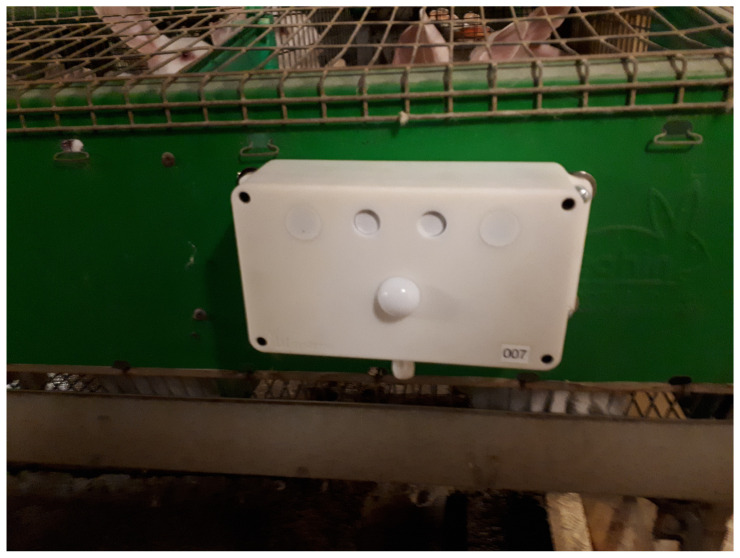
Installation of the device on the rabbit cages.

The devices were installed using magnets attached to the four corners of the case so that they could be directly attached to the cages at the rabbits’ level (Figure 5).

Data recorded with the six devices were organized into hourly values and processed to assess the differences among the monitoring points, using the generalized linear model procedure of IBM SPSS Statistics v 29. A pairwise comparison was used to indicate the significant differences among devices.

#### 2.3.2. Daily and Hourly Patterns of Environmental Factors in a Pig Farm

Recordings were carried out in the gestation room of a pig farm located in Visano (Brescia, Italy), where sows were raised in pens with partially slatted floors. The monitored room hosted the second phase of gestation (after the first 4 weeks from insemination) and consisted of 24 pens each of 23.4 m^2^ of total surface area and 9.9 m^2^ of slatted floor, housing up to 10 animals each.

The building used natural ventilation, with a window opening controlled with a unit based on temperature and humidity. In addition, fans were used for ventilation in the summer.

**Figure 6:**
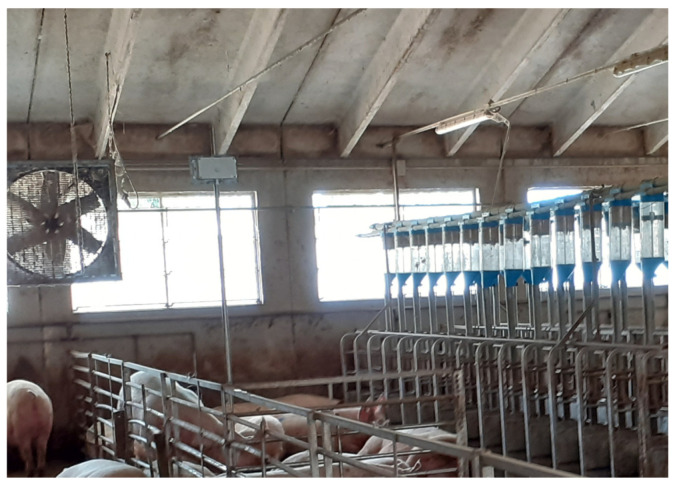
The device installed in the room for gestating sows.

Two N11 devices were installed in the central part of the room at an approximate height of 2.2 m from the floor (Figure 6). In this study, the reported data refer to the period from 13 June 2023 to 5 August 2023. The internal temperature, CO_2_, illuminance, and NH_3_ were specifically analyzed by comparing the averages of each hour of the day for the entire study period. The results were integrated with external temperatures obtained from a meteorological station located in Manerbio (Brescia, Italy), which is a part of the monitoring network of the Lombardy Region (www.arpalombardia.it, accessed on 16 November 2024).

#### 2.3.3. Seasonal Patterns and NH_3_ Emission in a Dairy Cow Farm

Monitoring was carried out in a livestock farm with 140 lactating cows (average milk yield 37 kg head^−1^ day^−1^) and a total of 290 heads from 5 September 2023 to 23 March 2024.

Three N11 devices were installed in the barn for lactating cows housed in three rows of cubicles with straw litter in a covered area of 1020 m^2^ and with an uncovered exercise area of 216 m^2^. An additional N11 device was placed outside the barn to record background concentrations together with a meteorological station (Meteosense 2.0, Netsense srl, Sesto Fiorentino, Florence, Italy).

**Figure 7:**
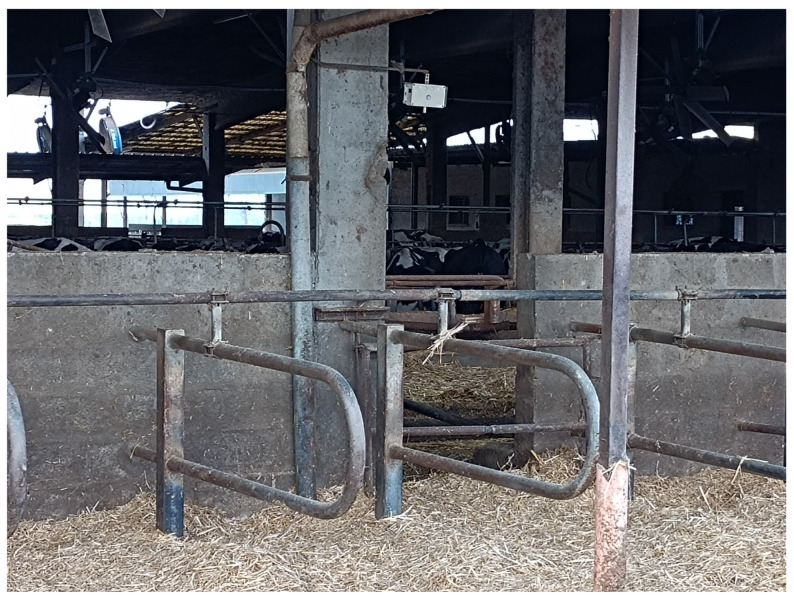
The device installed in the dairy farm.

The devices were attached with magnets to metal plates connected to the barn structure with clamps (Figure 7). The temperature, humidity, and concentrations of NH_3_ and CO_2_ were processed as average daily values.

The data collected with the N11 devices were used to estimate NH_3_ emissions using the methods reported in the VERA protocol for Livestock Housing and Management Systems [22]. The employed methodology was based on the use of CO_2_ as a trace gas. CO_2_ emissions were corrected for the ambient temperature and calculated according to the number of cows and their milk production level and energy intake. CO_2_ and NH_3_ concentrations inside and outside the barn were obtained from N11 device recordings. The data from the three devices were averaged.

NH_3_ emissions were obtained as follows:

(1) $$E_{NH3}=P_{CO2}\times \frac{\left[C_{NH3}\right]barn-\left[C_{NH3}\right]outside}{\left[C_{CO2}\right]barn-\left[C_{CO2}\right]outside}$$

where

*E_NH_*_3_ is NH_3_ emission (g h^−1^);
*P_CO_*_2_ is the CO_2_ produced by cows (g h^−1^);
*C_NH_*_3_ is the NH_3_ concentration inside and outside the barn (g m^−3^);
*C_CO_*_2_ is the CO_2_ concentration inside and outside the barn (g m^−3^).

Calculations were performed on an hourly basis to consider the fluctuating temperature in the barn and averaged over 24 h.

## 3. Results and Discussion

### 3.1. Uniformity of Environmental Conditions in a Rabbit Farm

Table 3 reports the average values of the various environmental factors measured by the six N11 devices throughout the entire monitoring period. The significant differences between the hourly values detected by the six control units are also reported in the table. The variability within the building was very high, especially for gas concentrations, namely NH_3_. In two measuring points (ID 4 and ID 6), the average daily values were higher than the 10 ppm limits outlined in the guidelines. The reported CO_2_ concentrations can be considered high despite not reaching the thresholds of 2500 ppm or being close to or over the value of 2000. On the contrary, temperature and humidity were in the acceptable range of values, with humidity close to a lower limit of 60%.

**Figure 8:**
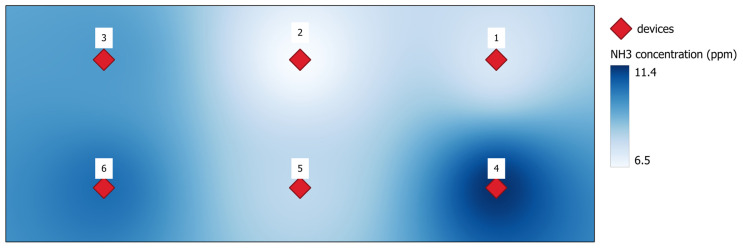
Distribution map of the average NH_3_ concentration values in the rabbit house shown in Figure 3. The numbers indicate the device ID.

Figure 8 shows a distribution map of the reported NH_3_ from the average values of each device and highlights an area of the room where ventilation was insufficient in keeping the gas concentration at levels lower than 10 ppm, which was indicated as the upper limit in guidelines for rabbit welfare [23].

The uneven concentration of gasses in the building is a consequence of the management of the ventilation system. In the winter period (the average external temperature in the monitoring period was 9.2 °C), most of the fans are stopped and only a minimum flowrate is maintained, especially during the night. Therefore, preferential air movement is established and in some zones of the building the gas concentration increases. A more distributed air inlet and a better control of the fans based on gas concentrations would improve the situation.

The results obtained in this experiment highlight the importance of monitoring the environmental parameters in rabbitries and how different devices might show critical points in the farm due to non-uniform ventilation or incorrect management. NH_3_ concentrations above 10 ppm were previously observed by other authors [24], but the average values observed by Calvet et al. [25] were below 7 ppm, confirming the significant variation among farms. Moreover, it was considered that the external environmental conditions could significantly affect internal concentration as farmers reduce the ventilation to a minimum in cold conditions to maintain an adequate temperature inside the building. This is confirmed by another experience that reported maximum concentration values of up to 20 ppm [25].

### 3.2. Daily Pattern in a Pig Farm

**Figure 9:**
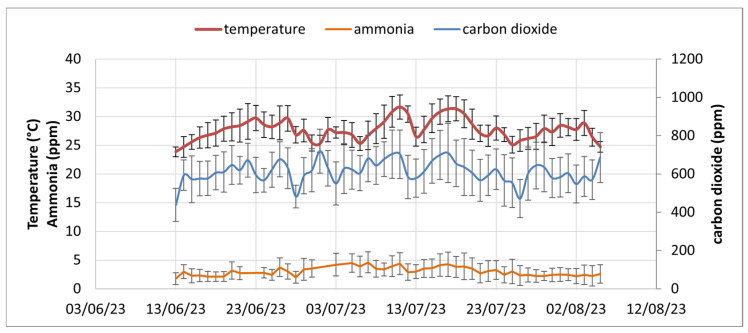
Daily average of temperature, CO_2_, and NH_3_ in the monitored piggery. Bars represent standard deviation.

The data obtained in the monitoring period made it possible to assess the over-time trends for the various environmental parameters and their compliance with the values indicated for animal welfare. The daily average values in Figure 9 show that there are no notable problems with CO_2_ and NH_3_, which remain below the threshold values indicated by the EFSA [8]. As a daily average, CO_2_ and NH_3_ reach a maximum of 720 ppm and 4.6 ppm compared to the threshold values of 3000 and 10 ppm, respectively. In contrast, the temperature is always very high, with maximum and average values of 31.7 °C and 27.7 °C, respectively. The thermoneutral zone for pregnant sows is generally considered to be between 16 and 21 °C [4,26], but some studies reported that sows prefer lower temperatures at the end of gestation (around 14 °C) [27,28].

In the monitored housing, it is evident that sows are under heat stress conditions, which may have significant adverse effects on animal welfare, such as production and reproduction [7].

As shown in Figure 9, the three considered parameters correlated with each other as expected. The correlation coefficient between temperature and carbon dioxide is 0.50 (*p* < 0.001), and the one between temperature and NH_3_ is 0.46 (*p* < 0.001).

These results highlight the need to improve the environmental conditions of farms by introducing cooling systems. Furthermore, the correlation found between gas concentration and temperature provides another element to consider when assessing emissions from farms under different climatic conditions.

**Figure 10:**
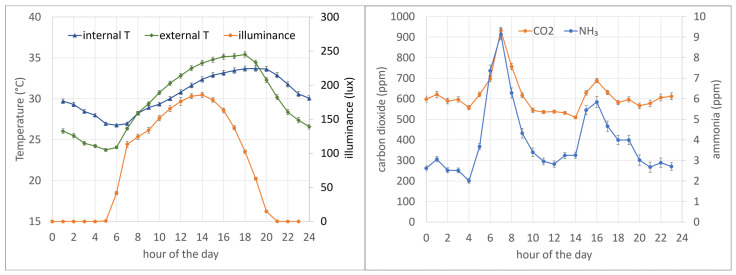
Average hourly values of internal and external temperature and illuminance (**left**) and CO_2_ and NH_3_ concentrations (**right**) recorded in a pig farm. Bars represent the standard error.

It is also interesting to consider how the daily averages are derived from a daily pattern with considerable variability. The analysis of the hourly average values of the considered factors showed that two peaks in NH_3_ and carbon dioxide concentrations occur during the day (Figure 10). These are at the feeding operations that are carried out at 7:15 a.m. and 4:30 p.m. It can be seen that the concentration of both NH_3_ and CO_2_ starts to increase from 5 a.m. alongside daylight and the animals waking up. The highest values occur at around 7 am when the feed is delivered. The afternoon peak is less pronounced and occurs at around 4 pm, which is the time of the second feed distribution.

The higher values in the morning could be attributed to increased animal activity at this time of the day, corresponding to light and temperature increases. However, there are no trends linked to temperature variations throughout the day. The lower activity in the afternoon is likely linked to the high temperatures (33–34 °C) at afternoon feeding time. These patterns are not unexpected and are common to other species [29].

### 3.3. Seasonal Patterns and NH_3_ Emissions in a Dairy Cow Farm

**Figure 11:**
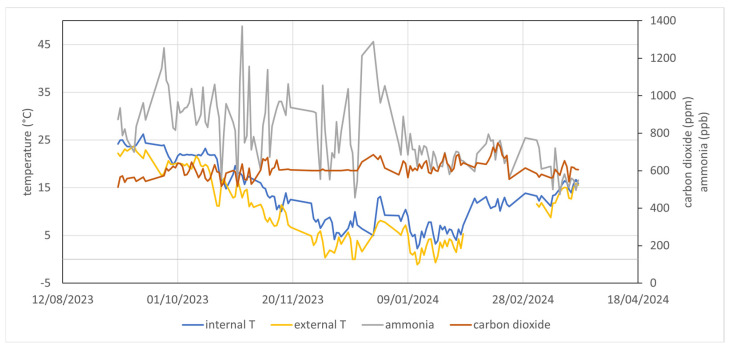
Daily average of temperature, NH_3_, and CO_2_ concentrations recorded in a dairy cow farm.

Figure 11 shows the trends in the indoor and outdoor temperatures and the carbon dioxide and NH_3_ concentrations.

The average daily temperature inside the barn was always slightly higher than the outside temperature. The average increase of 3 °C was due to the heat produced by the animals in the barn, but the temperature can be affected by the operation of the cooling system (fans in the resting area and sprinklers in the feeding area).

The NH_3_ concentration recorded has considerable variability but is very low. The average value was 0.8 ppm, with a range from 0.5 ppm to 1.4 ppm and a coefficient of variation of 23%. The CO_2_ concentration was more stable, with a mean value of 609 ppm and a coefficient of variation of 7%. The gas concentrations were dependent on the ventilation rate of the building, which, being open, was affected by the ambient conditions outside the building and the operation of the ventilation system. Thus, this kind of variability is expected [30,31].

**Figure 12:**
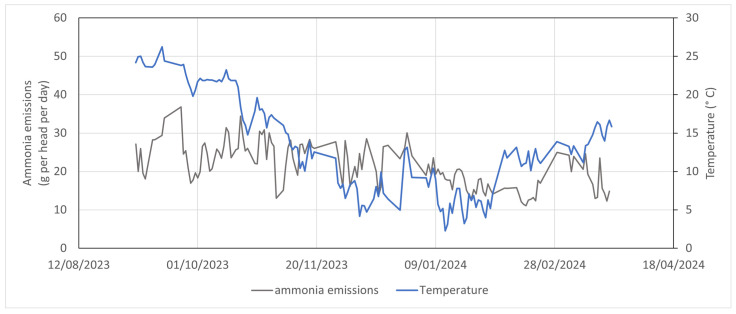
Daily values of NH_3_ emissions and temperature in the dairy cow barn.

The results obtained from the estimate of NH_3_ emissions are reported as daily values in Figure 12. The reported NH_3_ emissions of dairy cows varied between 30 and 45 g per animal a day. These values are in line with those reported by Groot Koerkamp et al. [32] and are affected by seasonality and temperature. The correlation with the temperature inside the barn is not significant and the effect of other factors influencing emissions should be investigated. It should also be noted that the method used was affected by numerous uncertainties related to the accuracy of the measurement system, the representativeness of the measurements in the barn, and some assumptions of the method. However, the possibility of continuously collecting data allowed us to understand the trend in emissions over time with equipment that can be easily installed in livestock farms. The validity of the method must also be verified in different conditions by comparing it with reference methods.

### 3.4. Applicability and Limitations of the Device in Livestock Housings

The results obtained highlight that the use of devices based on low-cost sensors and with IoT connection, like the one described in this paper, can be considered adequate for use in animal husbandry. The findings of this work confirm those of other authors in relation to gas concentrations with low-cost devices [14,15,16], reaffirming the importance of expanding the use of multi-sensor devices for environmental conditions monitoring on livestock farms, supporting the conclusions of Danev et al. [17].

The cost of these multi-parameter devices (<USD 2500) allows their use in livestock facilities to monitor environmental conditions, even at different points in the facility, which is not practical with more precise instruments that have a significantly higher cost (USD 60,000), such as FTIR. Moreover, the availability of an online dashboard to view and download the recorded data makes monitoring activity and subsequent data processing simpler, eliminating the need for intervention in the farms.

We also demonstrated the usefulness of the data recorded with this type of device in assessing suitable conditions for animals. These data can be used to identify reduced ventilation uniformity, highlighted by elevated CO_2_ or NH_3_ concentrations inside the building. The recordings of daily patterns of CO_2_ and NH_3_ can reveal concentration peaks caused by farm operations, such as feed delivery and manure removal. Moreover, correlations between temperature and gas concentrations can be obtained, and continuous monitoring of concentrations might also provide an estimate of the emissions of gasses such as NH_3_.

However, it should be emphasized that the use of low-cost sensors should be carefully considered regarding the accuracy of measurements. In particular, as far as CO2 is concerned, the measurements provide useful information for the control of the ventilation system, but they must be carefully evaluated for the purposes of emission estimation, as pointed out by D’Urso et al. [14]. In addition, it should be considered that some sensors may have a time-dependent loss of precision and may require periodic recalibration or replacement.

## 4. Conclusions

This study outlined an IoT device to be used for the environmental monitoring of livestock housing, providing three case studies of its implementation with different purposes in livestock farms.

Monitoring environmental conditions with multi-sensor devices can support farmers to improve environmental performance and increase animal welfare and production quality. For this purpose, further research is needed to analyze the operation of these devices over the long term to assess the effects over time in barn conditions and evaluate possible cross-interference with moisture and other gasses.

In addition, for the purpose of continuous monitoring in housing facilities, it is necessary to develop a system for analyzing data collected at different points in the facility to highlight information useful for optimal animal management, potentially incorporating artificial intelligence methodologies.

The systematic use of these devices on livestock farms can enable a detailed analysis of the microclimatic and air quality conditions in which animals live and thus significantly contribute to improving animal welfare.

## Figures and Tables

**Table 1:** Sensors installed in the N11 device and their main characteristics.

Sensor	Type	Technology	Range	Accuracy
Temperature	Digital	CMOS	−10–+60 °C	±0.2 °C
Relative humidity	Digital	CMOS	0–100%	±2%
Sound pressure	Analog	MEMS	30–130 dBA	±2%
Illumination	Analog	ALS Photodiode	0–128 klux	±30%
H_2_S	Analog	Electrochemical	0–10 ppm	±10%
NH_3_	Analog	Electrochemical	0–100 ppm	±10%
CO_2_	Digital	NDIR	400–10,000 ppm	±30 ppm/±3%
VOC (VOCs)	Analog	CMOSense	500–10,000 ppb	±15 ticks /0.03%
VOC (NOx)	Analog	CMOSense	50–650 ppb	±50 ticks/0.1%
PM_1_, PM_2.5_	Digital	Laser scattering	0–1000 µg m^−3^	±10 µg m^−3^
PM_4_, PM_10_	Digital	Laser scattering	0–1000 µg m^−3^	±25 µg m^−3^

**Table 2:** Average and standard deviation of the errors of the sensors.

Sensor	Average Error		Average Standard Deviation	
	Absolute	Relative	Absolute	Relative
Temperature	0.04 °C	0.21%	0.05 °C	0.27%
Relative humidity	0.12%	0.17%	0.15%	0.22%
Sound pressure	0.54 dBA	0.79%	0.71 dBA	1.02%
CO_2_	15.3 ppm	1.76%	20.1 ppm	2.31%
PM 2.5	0.31 µg m^−3^	2.46%	0.41 µg m^−3^	3.28%
NH_3_	0.13 ppm	2.69%	0.09 ppm	2.08%
H_2_S	0.04 ppm	1.38%	0.05 ppm	1.69%

**Table 3:** Average daily values and significance of the differences among devices for the monitored environmental factors. Different letters indicate a statistical difference (*p* < 0.01) among devices.

Device	Temp.		UR		NH_3_		CO_2_		H_2_S		Sound		PM_2.5_	
	°C		%		ppm		ppm		ppm		dbA		µg m^−3^	
ID1	21.9	e	62.5	c	7.3	e	2287	a	0.00	d	57.7	e	17.2	bc
ID2	22.1	d	59.8	d	6.5	f	1998	d	0.00	d	58.8	c	18.6	ab
ID3	22.3	c	63.2	b	9.5	c	2272	a	0.00	d	58.7	c	16.5	c
ID4	22.4	b	62.9	b	11.4	a	2203	b	0.01	c	58.3	d	17.8	ab
ID5	22.5	a	58.1	e	7.9	d	1945	e	0.04	a	59.0	b	17.1	bc
ID6	21.6	f	66.0	a	10.4	b	2125	c	0.02	b	59.3	a	17.5	b

## Data Availability

The original contributions presented in the study are included in the article, further inquiries can be directed to the corresponding authors.
